# Stem cell-derived retinal pigment epithelium cell therapy: Past and future directions

**DOI:** 10.3389/fcell.2023.1098406

**Published:** 2023-03-30

**Authors:** Landon J. Rohowetz, Peter Koulen

**Affiliations:** ^1^ Vision Research Center, Department of Ophthalmology, School of Medicine, University of Missouri—Kansas City, Kansas City, MO, United States; ^2^ Department of Biomedical Sciences, School of Medicine, University of Missouri—Kansas City, Kansas City, MO, United States

**Keywords:** retina, retinal pigment epithelium, embryonic stem cell (ES cell), iPSC (induced pluripotent stem cell), Stargardt disease, age-related macular degeneration (AMD)

## Abstract

The eyes are relatively immune privileged organs, making them ideal targets for stem cell therapy. Researchers have recently developed and described straightforward protocols for differentiating embryonic and induced pluripotent stem cells into retinal pigment epithelium (RPE), making diseases affecting the RPE, such as age-related macular degeneration (AMD), viable targets for stem cell therapy. With the advent of optical coherence tomography, microperimetry, and various other diagnostic technologies, the ability to document disease progression and monitor response to treatments such as stem cell therapy has been significantly enhanced in recent years. Previous phase I/II clinical trials have employed various cell origins, transplant methods, and surgical techniques to identify safe and efficacious methods of RPE transplantation, and many more are currently underway. Indeed, findings from these studies have been promising and future carefully devised clinical trials will continue to enhance our understanding of the most effective methods of RPE-based stem cell therapy, with the hope to eventually identify treatments for disabling and currently incurable retinal diseases. The purpose of this review is to briefly outline existing outcomes from initial clinical trials, review recent developments, and discuss future directions of clinical research involving stem-cell derived RPE cell transplantation for retinal disease.

## Background

The use of stem cells in research and therapy has been the subject of controversy since the isolation of human embryonic stem cells (hESCs) in 1998 at the University of Wisconsin-Madison (Thomson et al., 1998). At that time, scientists were able to create a cell line from human blastocyst-derived cells that proved to be immortal and pluripotent. While promising for science insofar that researchers had discovered a supply of cells that could be easily grown and stimulated to differentiate into various cell types, there were obvious and significant ethical concerns regarding the use of cells obtained from human embryos. Indeed, in 2001, shortly after the discovery of hESCs, the United States government halted funding and support for the creation of further hESCs for research purposes. While research involving previously obtained hESCs was authorized, the restriction ultimately limited availability of hESC lines to less than one hundred, impacting the direction of research approaches (Shevde, 2012).

However, in 2006, researchers in Japan discovered induced pluripotent stem cells (iPSCs) by transforming mouse fibroblasts into pluripotent stem cells (Takahashi and Yamanaka, 2006). Less than a year later, iPSCs were formed from human cells (Takahashi et al., 2007).

Induced pluripotent stem cells possess two significant advantages over embryonic stem cells (ESCs). First, iPSCs eliminate the need for ESCs as they are reprogrammed from mature adult somatic cells. Second, iPSCs can be made autologously, meaning that each individual can theoretically have their own iPSC line. The disadvantages include a slow rate of reprogramming somatic cells into iPSCs, the possibility of introducing mutations into target cells by transcription factors required for reprogramming, and the potential for tumor generation due to the expression of oncogenes triggered intentionally and unintentionally by viruses used to genomically alter cells. Indeed, c-Myc, an oncogene found in many cancers, was initially one of four genes required to create iPSCs (Takahashi and Yamanaka, 2006). However, in 2008, researchers demonstrated the ability to create iPSCs without c-Myc, albeit at a slower rate (Nakagawa et al., 2008; Wernig et al., 2008). Furthermore, researchers showed that iPSCs can be created without the use of viruses through the utilization of plasmids that can eventually be removed and, most recently, proteins, chemicals, and miRNA molecules that mimic the actions of transcription factors without the associated mutagenic potential (Soldner et al., 2009; Hu, 2014).

Despite the advances made with iPSCs, and possibly because of the high cost and time needed for their development, interest in using ESCs for research remained high. Indeed, in 2009, restrictions on federal funding for human stem cell research changed and the Food and Drug Administration approved the first clinical trial using hESCs (Alper, 2009; Shevde, 2012). Since then, ESC-based treatments have been proposed for a diverse range of disorders including myocardial infarction, stroke, amyotrophic lateral sclerosis, Pelizaeus-Merzbacher disease, Alzheimer’s disease, and Parkinson’s disease (Schwartz et al., 2015; Aznar and Tudela, 2016).

The eyes are of particular interest in stem cell-based treatments. The subretinal space is protected by the blood-ocular barrier, resulting in a muted immune response to foreign antigens compared with other sites in the body. As such, the eye is a relatively safe target for novel cell-based treatment approaches such as those involving ESCs. Retinal pigment epithelial (RPE) cells are particularly suitable substrates for transplantation, as these cells can be differentiated and reproduced relatively easily as compared to other types of cells. Furthermore, optical coherence tomography and microperimetry enable researchers and clinicians to precisely measure the structural and functional success of treatment postoperatively (Kashani et al., 2018; Mehat et al., 2018). Given these factors, in combination with the prevalence of diseases affecting RPE such as age-related macular degeneration (AMD), Stargardt disease, and retinitis pigmentosa, the development of safe and effective means of RPE stem cell transplantation has become a desirable achievement in ophthalmology and vision research. The purpose of this review is to broadly illustrate outcomes of prior clinical trials, review recent developments, and discuss future directions of clinical research involving stem-cell derived RPE cell transplantation for retinal disease.

## Initial studies

In 2010, researchers began the second clinical trial involving hESCs and the first involving hESC-derived RPE, this time targeting dry AMD and Stargardt disease (Schwartz et al., 2012). Patients received a suspension of hESC-derived RPE cells in the subretinal space. The results of this study were encouraging insofar that the investigators observed increased pigmentation in 13 of 18 patients and improved visual acuity in 10 of 18 patients after transplantation of RPE cell suspensions into the subretinal space, although no correlation was observed between vision improvement and increased subretinal pigmentation (Schwartz et al., 2015). Adverse events related to the transplanted tissue were not observed (Schwartz et al., 2012; Schwartz et al., 2015; Song et al., 2015; Brant Fernandes et al., 2022).

With the advent of iPSCs and the potential of adult stem cells, some researchers transitioned their focus to iPSC-based models and treatments, which circumvent the need for cells derived from a human embryo. While overall there are currently more active clinical studies involving hESCs than iPSCs, there are more iPSC-based clinical studies in the recruitment phases, possibly reflecting the gradual transition to a predominantly iPSC-based approach. Translation and use of the findings from hESC studies provides significant potential to guide further iPSC-based inquiries and approaches.

While the majority of therapeutic research with iPSCs has involved *in vitro* and animal models, the first clinical trial with iPSCs was started in 2014 and in that same year a patient with neovascular AMD received the first iPSC-based treatment for any indication (Mandai et al., 2017). A second transplant was considered in this pilot study but was withheld due to the detection of genomic alterations (Mandai et al., 2017). Although the patient’s vision did not improve, the treatment was considered a groundbreaking success insofar that it demonstrated the feasibility and safety of iPSC transplantation in retinal disease, providing evidence and a stimulus for further iPSC-based retinal research. Indeed, in 2019, investigators at the NEI illustrated their success in transplanting mutation-free iPSC-derived RPE patches from AMD patients into pigs and rodents with models of AMD. A corresponding clinical trial using this protocol began in 2020 (Sharma et al., 2019; Wiley, 2020). First reports of trials using hESC-derived RPE cells and a similar approach (Kashani et al., 2020) indicate positive outcomes with respect to safety, tolerability and efficacy (Kashani et al., 2021).

## Recent findings

There are two prevailing methods for transplanting RPE cells into the eye. The first and more traditional method involves the injection of a suspension of stem cells into the subretinal space. As alluded to above, in 2015, Schwartz and colleagues reported on the transplantation of suspensions of hESC-derived RPE into nine patients with dry AMD and nine patients with Stargardt disease. Visual acuity improved in ten patients and no serious safety issues related to the transplantation were reported (Schwartz et al., 2015). Similarly, in 2015, Song and colleagues reported results of a similar protocol involving two patients with AMD and two patients with Stargardt disease. Three of the four patients experienced improvements in visual acuity and there were no adverse events related to the transplanted cells (Song et al., 2015). In 2018, Mehat et al. demonstrated the findings from 12 patients with advanced Stargardt disease treated with hESCs *via* the suspension approach. The authors found no evidence of functional visual benefit as measured *via* microperimetry, visual acuity or subjective questionnaire. There were areas of increased pigmentation at the site of transplantation. However, in some instances, these areas were associated with reduced macular sensitivity, implying that while subretinal hyperpigmentation may reflect the presence viable transplanted cells, it also may represent released pigment from dead cells (Mehat et al., 2018). The authors hypothesized that the lack of functional benefit may have been secondary to the advanced stage of disease in the study population and that further studies may seek to include patients with milder disease. A recent phase I clinical trial using subretinal injection of hESC-RPE cells in suspension reported similar outcomes with a non-significant improvement in visual acuity (Brant Fernandes et al., 2022).

The second method of transplantation involves implanting cells as a monolayer on a scaffold. While the traditional suspension method has a lower risk of complications such as retinal detachment and subretinal hemorrhage, cells usually do not form a polarized monolayer, may not cover atrophic areas sufficiently, and display inferior survival and functional outcomes compared to scaffolds (Tezel and Del Priore, 1997; Hu et al., 2012). Da Cruz and colleagues reported on the first transplantation using an RPE patch in two patients with neovascular AMD. These patients demonstrated hyperpigmentation, functioning RPE phagocytosis, and visual recovery measured by visual acuity, macular sensitivity, and reading speed. There were no serious adverse events related to the RPE patch (da Cruz et al., 2018). In the same year, Kashani and colleagues reported the results from transplantation of an RPE patch into four eyes with dry AMD, demonstrating an improvement in visual acuity in one eye and fixation improvements in two eyes. Mild to moderate self-resolving subretinal hemorrhages occurred in all patients while one patient experienced a more significant subretinal hemorrhage that resolved with administration of a single dose of bevacizumab (Kashani et al., 2018). Furthermore, in 2019, researchers at the NEI illustrated a protocol for functionally validating a clinically compatible autologous human iPSC RPE manufacturing process using biodegradable patches derived from multiple patients. They also compared the safety and efficacy of these RPE patches to cell suspensions transplanted in pigs and rats, demonstrating superior integration and functionality with the patches (Sharma et al., 2019). Recent studies using decellularized human amniotic membrane as a scaffold for hESC-derived RPE cell monolayers showed mixed results with a failure to form a consistent RPE layer and basement membrane (Daniele et al., 2022). The authors hypothesized that this failure occurred because of a mismatch between surface molecules on the amniotic membrane and RPE cell receptors. Further research is necessary to evaluate the relationship between these variables and the impact of other factors such as donor characteristics and cryopreservation methods.

In addition to being classified as induced pluripotent or embryonic, stem cells can be characterized as autologous or allogenic. Autologous stem cells are those derived from the patient’s own tissue while allogenic stem cells are from another human. While the eye is a relatively immune-privileged tissue, rejection is possible in diseases in which the RPE and thus the blood-retinal barrier is affected (West et al., 2020). Since autologous stem cells have the antigenic makeup of the patient receiving the therapy, they carry less potential for an immune response. Indeed, in 2014, Kamao et al. demonstrated the ability to consistently produce hiPSC RPE cell sheets under good manufacturing practices and subsequently transplant these sheets into animals. The authors were able to do so without the use of an artificial scaffold, which may be a source of inflammation and impaired physiologic function, instead using extracellular deposits to maintain the integrity of the RPE cell monolayer. Furthermore, the authors found no cancer-causing mutations in their study (Kamao et al., 2014). One human subject was enrolled with successful implantation and no adverse events but no improvement in visual acuity or retinal sensitivity after 4 years, likely secondary to preexisting photoreceptor damage and scarring (Mandai et al., 2017; Takagi et al., 2019). While promising insofar that the graft in the aforementioned study survived for 4 years, it was ultimately determined that the production of autologous iPSC-based tissue for transplantation was cost- and resource-prohibitive and future directions would focus on the large-scale production and testing of pre-validated allogenic HLA-matched iPSC cells (Sugita et al., 2016; West et al., 2020). Indeed, investigators recently demonstrated the ability to produce and transplant allogenic hiPSC-derived RPE cells and allografts without inducing a T-cell response or rejection in MHC-matched animals (Sugita et al., 2016).

However, as alluded to above, in 2019, researchers at the NEI outlined a protocol for the consistent and efficient production of autologous human-derived iPSC RPE patches using good manufacturing practice protocols that can be safely transplanted into rodents and pigs with therapeutic effects (Sharma et al., 2019). A phase I/IIa clinical trial using this protocol to treat geographic atrophy associated with AMD began in 2020 (Wiley, 2020). Indeed, both allogenic and autologous stem cells may have a role in RPE transplantation. As such, further research using both cell types is warranted to evaluate the safety and practicality of both forms of transplantation. With advances in molecular profiling of stem cell-derived RPE cells (Petrus-Reurer et al., 2022), safety and efficacy measures are improving, thereby, further supporting the feasibility of transplantation approaches.

In addition to cell source and substrate, the surgical method of transplantation is an important variable to evaluate, as it is often a source of adverse events. While the traditional approach involves a *pars plana* vitrectomy and retinotomy, in 2014, Ho et al. demonstrated the ability to perform transplantation without these procedures. They employed an *ab externo* approach involving subretinal cannulation with subsequent visual acuity improvements in transplanted patients. However, a high incidence of complications occurred with this approach, including retinal detachment and retinal perforation (Ho et al., 2017). More recently, in 2020, Riemann and colleagues demonstrated data from two patients who received transplantation *via* a revised *ab externo* approach using suprachoroidal cannulation, with promising safety and efficacy results, including no instances of retinal detachment or retinal perforation (Riemann et al., 2020). Indeed, further research is needed to identify the most non-invasive yet effective surgical techniques for retinal stem cell transplantation.

Artificial scaffolds, while potentially a source of RPE dysfunction themselves, aid in delivery of the patch and are needed to maintain integrity of the polarized RPE monolayer. Scaffolds also provide support to RPE cells, as age-related changes in Bruch’s membrane, the interface between the choroid and the RPE, may hinder its natural ability to support transplanted RPE survival and differentiation (Gullapalli et al., 2004; Gullapalli et al., 2005). Many groups are evaluating the use of various materials including parylene and polyesters such as polyethylene terephthalate, poly (lactic-co-glycolic acid), polycaprolactone, and poly-L-lactic acid as scaffolds for RPE sheets (Lu et al., 2012). These scaffolds would act to maintain cell integrity and would prevent interaction with native Bruch’s membrane. As an alternative, some are also evaluating the use of temporary, degradable scaffolds that would act to facilitate RPE cell attachment to native Bruch’s membrane while limiting ongoing inflammation (Liu et al., 2014; Hotaling et al., 2016; Sharma et al., 2019; Wiley, 2020). Other groups have investigated the use of additional substances such as amniotic membrane and femtosecond laser intrastromal lenticule extraction-derived lenticules, in addition to manufacturing sheets without a scaffold using materials such as peptide-modified alginate hydrogels (Table 1) (Brant Fernandes et al., 2022; Mandai et al., 2017; Gu et al., 2019; Ben M'Barek et al., 2017; Soroushzadeh et al., 2022).

**TABLE 1:** Materials used as scaffolds for the transplantation of stem cell-derived retinal pigment epithelial cell monolayers.

Polyethylene terephthalate
Polyethylene terephthalate
Parylene
Poly (lactic-co-glycolic acid)
Polycaprolactone
Poly-L-lactic acid
Amniotic membrane
Femtosecond-derived lenticule

In addition to RPE cells, investigators have evaluated the safety and efficacy of non-RPE cell transplantation within the eye. In 2018, Zhang and colleagues demonstrated closure of refractory macular holes in six of seven patients treated with umbilical cord-derived mesenchymal stem cells (UC-MSCs) or exosomes derived from UC-MSCs, with an inflammatory response in one patient (Zhang et al., 2018). In 2020, Kahraman and Oner injected UC-MSCs into the suprachoroidal space of 124 eyes with retinitis pigmentosa and demonstrated improvements in visual acuity, visual field, and electroretinogram parameters at 6 months with no serious adverse events (Kahraman and Oner, 2020). In the same year, Zhao and colleagues demonstrated improved visual acuity in 91% of retinitis pigmentosa patients treated with intravenous UC-MSCs in addition to a significant improvement in NEI Visual Function Questionnaire scores and no serious adverse events (Zhao et al., 2020). Wharton’s jelly-derived mesenchymal stem cells have also demonstrated effectiveness in improving visual acuity, visual field, and outer retinal thickness in patients with retinitis pigmentosa when administered in the sub-tenon space (Ozmert and Arslan, 2020).

Furthermore, in 2018, Gu and colleagues demonstrated improvements in visual acuity without any significant adverse events in patients with non-proliferative diabetic retinopathy after treatment with intravenous bone marrow-derived mesenchymal stem cells (Gu et al., 2018). In 2020, Sung and colleagues performed sub-tenon transplantation of human placenta-derived mesenchymal stem cells and demonstrated and increase in visual acuity in all patients and an increase in the expression of Tuj1 and Gfap, suggesting a neuroprotective effect on retinal ganglion cells and the optic nerve. There were no significant adverse effects at 12 months (Sung et al., 2020). In 2021, Tuekprakhon and colleagues demonstrated stability of visual acuity, visual field, and central subfield thickness between 1.5 and 7 years after treatment of 14 patients with advanced stage retinitis pigmentosa with bone marrow-derived mesenchymal stem cells, with one severe but manageable adverse event (Tuekprakhon et al., 2021). Wiącek and colleagues demonstrated improvements in visual acuity, visual field, and electroretinogram parameters after intravitreal injection of bone marrow-derived lineage-negative cells in 30 patients with retinitis pigmentosa although 3 cases of local tractional retinal detachment were observed (Wiacek et al., 2021). Retinal detachments were suspected to occur due to the susceptibility of retinitis pigmentosa patients to vitreoretinal junction pathologies. The authors suggest that their rate may be decreased by maintaining RPE integrity during cell administration (Wiacek et al., 2021). Improvements in visual acuity have also been observed in AMD patients treated with subscleral injection of autologous adipose-derived stem cells (Limoli et al., 2014; Limoli et al., 2016).

## Future directions

Functional outcomes in clinical trials thus far have been relatively limited as expected due to the advanced stage of disease and poor baseline vision of the study populations required for phase I and II studies. However, safety results have been promising insofar that, while adverse events have arisen, none have been directly related to the transplanted tissue. Indeed, many of the adverse events were secondary to the surgeries themselves or the immunosuppression required for treatment. With the advent of less invasive surgical techniques, more precise HLA matching, and more efficient autologous differentiation and validation protocols, a reduction in the incidence of adverse events is likely.

There are currently at least ten active phase I/II clinical trials using hESCs to treat retinal disease and at least two active preclinical trials using iPSCs. Active iPSC studies include those investigating the manufacturing of hiPSC RPE from patients with Best disease and the transplantation of iPSC RPE to repair degenerative vessels in diabetic retinopathy.

Eventually, phase III studies will begin, requiring larger patient populations and multi-centered designs. As such, logistical factors including the development of cryopreservation techniques that can be easily employed on a large scale and in a diverse range of settings will be essential. Developing such processes now will promote further refinement of these protocols to ultimately increase accessibility for a wide range of patient populations and demographics. This is particularly important given the extensive prevalence of retinal disease such as AMD.

## Conclusion

The studies referenced above provide promise for the future of RPE-based stem cell therapy and represent the foundation for a new approach to retinal disease. However, many questions remain, which will likely be answered in the near future. Further comparative study will reveal the most appropriate cell types for transplantation including autologous or allogenic and induced pluripotent or embryonic. Given the widespread need for treatment of retinal disease, future study will also determine the safest, most effective, and practical variables in RPE stem cell therapy including transplant type, surgical methods, and cryopreservation techniques. Indeed, the answers to these questions are critical foundations for the development of treatments that have the potential to significantly decrease morbidity and improve quality of life for patients suffering from disabling and currently irreversible forms of retinal damage.

## Abbreviations

AMD, age-related macular degeneration; ESC, embryonic stem cell; hESC, human embryonic stem cell; hiPSC, human induced pluripotent stem cell; iPSC, induced pluripotent stem cell; NEI, National Eye Institute; RPE, retinal pigment epithelium; UC-MSC, Umbilical cord-derived mesenchymal stem cells.
